# The pivotal role of mammalian target of rapamycin inhibition in the treatment of patients with neuroendocrine tumors

**DOI:** 10.1002/cam4.742

**Published:** 2016-08-18

**Authors:** Alexandria T. Phan, Bhuvanesh Dave

**Affiliations:** ^1^ Houston Methodist Hospital Cancer CenterHoustonTexas

**Keywords:** Combination therapy, mammalian target of rapamycin, neuroendocrine tumor, pNET, signaling pathways

## Abstract

Significant advances have been made toward understanding the biology of neuroendocrine tumors (NET) in terms of defining prognosis and improving clinical management; however, many unmet needs remain. The treatment landscape for NET has evolved, with the approval of the targeted agents everolimus and sunitinib for the treatment of advanced pancreatic NET in 2011 followed by the approval of everolimus for the treatment of advanced nonfunctional gastrointestinal and lung NET in 2016. Mammalian target of rapamycin (mTOR) and components of the mTOR pathway play pivotal roles in NET pathogenesis. Effects of the mTOR inhibitor everolimus have been well documented in preclinical and clinical studies, both as monotherapy and combination therapy. mTOR inhibition as backbone therapy within the NET treatment landscape is a focus of continuing research, which includes evaluation of the growing armamentarium of approved and investigational agents as potential combination partners. Data evaluating the clinical benefits of agents targeting mTOR and related pathways (alone and in combination) in the treatment of patients with NET continue to increase. Many of the findings to date are encouraging.

## Introduction

Encouraging advances have been made toward understanding the biology of neuroendocrine tumors (NET) in terms of defining prognosis and improving clinical management. Per the Surveillance, Epidemiology, and End Results database, annual adjusted incidence of NET in the United States increased from 1.09 per 100,000 in 1973 to 5.25 per 100,000 in 2004 1. This observed increase is thought to be attributable, at least in part, to improved diagnostic approaches 2. NET are a group of heterogeneous neoplasms arising from islets of Langerhans cells of the pancreas (pancreatic NET [pNET]) or from the enterochromaffin cells of the thorax or gastrointestinal tract, some of which might cause symptoms because of hormone secretion (functional NET) 3, 4, 5.

NET are divided into clinically relevant groups based primarily on embryonic origin of primary site (foregut, midgut, and hindgut), histology (per World Health Organization [WHO] criteria, based on 2 proliferative indices—Ki67 and mitotic figures counted on microscope), and hormonal functionality (functional vs. nonfunctional tumors) 2, 6. Well‐differentiated NET (WD‐NET) are generally of low or intermediate grade and are more indolent 6, whereas poorly differentiated NET are generally of high grade and are aggressive 1, 6. While WD‐NET of the lung and gastrointestinal tract and WD‐NET of the pancreas (pNET) may share histologic characteristics such as proliferation indices, their biologic markers vary and response to therapeutic agents markedly differs; these tumors should therefore be examined separately in clinical trials 7, 8, 9.

Prognosis for patients with NET depends on three very important clinical characteristics—histology, stage, and primary disease site 1. Surgical resection offers the best chance for durable remission; however, many patients with NET present with advanced disease 8. Since 2009, results of several pivotal clinical trials have led to approval by the US Food and Drug Administration (FDA) of several agents for NET, specifically WD‐NET. While treatment options for these patients continue to expand, these agents have not generated robust tumor cytoreduction or improved overall survival (OS). Novel agents, sequencing, and combination therapies remain an active area of clinical research that might help improve patient outcomes. The somatostatin and vascular endothelial growth factor (VEGF) pathways are relevant and meaningful targets in NET, but the most promising and innovative target for developing therapeutic strategies for NET is the mammalian target of rapamycin (mTOR) pathway.

### Targeted therapies for clinical management of advanced NET

The aim of therapy for patients with advanced NET is to achieve tumor control through eradicating or stabilizing disease, prolonging survival, and relieving the symptoms of functional tumors, while improving patients’ overall functioning and maintaining their quality of life 5.

In the past, standard therapies for patients with advanced pNET included chemotherapeutic agents such as streptozocin‐based regimens and somatostatin analogs (SSAs) 10, 11, whereas therapy for midgut NET was focused specifically on symptom control. Antitumor effects of SSAs have been demonstrated in two phase 3, randomized, double‐blind, placebo‐controlled studies—PROMID (octreotide long‐acting repeatable [LAR]; Novartis Pharmaceuticals Corporation, East Hanover, NJ) 12 and CLARINET (lanreotide Autogel®/Depot; Ipsen Biopharmaceuticals Inc., Basking Ridge, NJ) 13. Despite many differences between these studies with regard to patient populations (e.g., midgut NET in PROMID vs. gastroenteropancreatic NET in CLARINET), as well as differences in assessment of tumor progression (bidimensional WHO criteria in PROMID vs. unidimensional Response Evaluation Criteria In Solid Tumors [RECIST], version 1.0 in CLARINET) 12, 13, findings from both studies are aligned in demonstrating clinically relevant antiproliferative effects with SSAs in patients with NET. Octreotide LAR is approved by the FDA for symptom control, particularly severe diarrhea/flushing episodes and symptoms of VIPomas 14, whereas lanreotide was recently FDA‐approved for patients with unresectable, well‐ or moderately differentiated, locally advanced, or metastatic gastroenteropancreatic NET to improve progression‐free survival (PFS) 15. Regulatory approval based on the antiproliferative activity of octreotide LAR demonstrated in PROMID varies by country; both octreotide LAR and lanreotide are approved in Europe for antitumor control as well as relief of symptoms associated with functional NET 14, 15, 16, 17. Since 2011, two targeted agents, the VEGF–tyrosine kinase inhibitor (TKI) sunitinib (Pfizer Inc, New York, NY) and the mTOR inhibitor everolimus (Novartis Pharmaceuticals Corporation), have been approved in the United States and the European Union 18, 19, 20, 21. Each agent demonstrated improvement versus placebo in a phase 3 trial 22, 23, leading to FDA approval for treatment of patients with progressive, unresectable, locally advanced, or metastatic pNET 18, 19. In 2016, demonstrated efficacy in the phase 3 RADIANT‐4 trial led to the expansion of everolimus’ indication to include patients with progressive, unresectable, locally advanced or metastatic, nonfunctional gastrointestinal and lung NET 18. This review focuses on mTOR inhibition and its role as a backbone therapy within the NET treatment landscape.

### Alterations in the mTOR pathway

**Figure 1:**
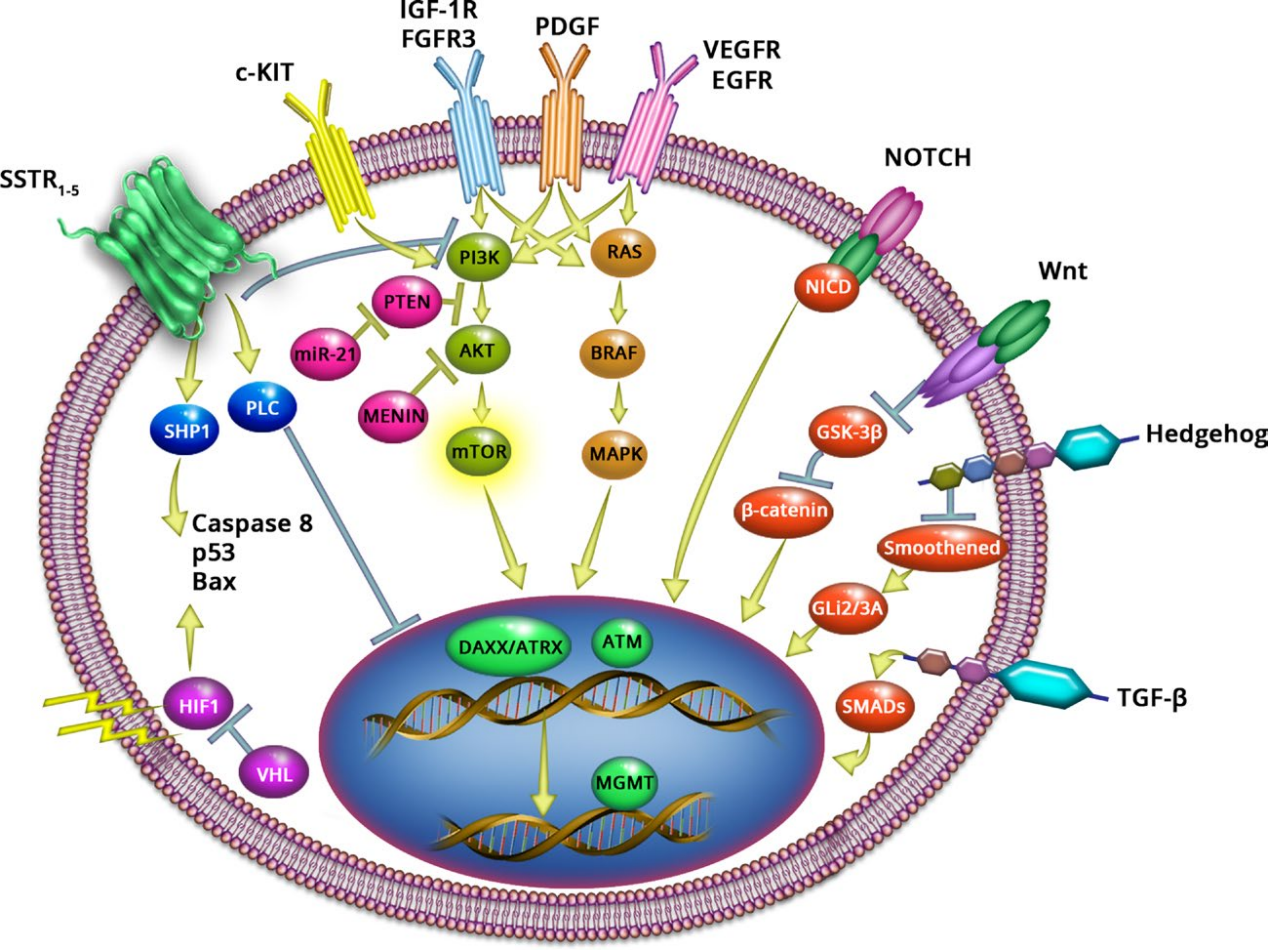
Upstream and downstream signaling in the mTOR pathway.

mTOR regulates essential cellular signaling pathways and plays a role in coupling growth stimuli to cell‐cycle progression 24. mTOR is a serine/threonine kinase composed of two distinct protein complexes, mTOR complex 1 (mTORC1) and 2 (mTORC2) 25, 26, 27. Various upstream intracellular and extracellular signals activate mTOR, including nutrients and receptor tyrosine kinases (RTKs) such as the epidermal growth factor receptor (EGFR), insulin‐like growth factor receptor (IGFR), and VEGF receptor (VEGFR) families (Fig. 1) 28, 29. mTOR acts as a key mediator by integrating signaling pathways associated with cell growth and proliferation, cell survival, tumorigenesis, angiogenesis, and cell metabolism 26, 30. Of note, aberrant activation of this pathway has been detected in many types of neoplasms, including NET 31, 32, 33, 34, as a result of excessive signaling by upstream cytokines and growth factors 24, 26, 27, 28, 29, 35. The binding of upstream RTKs by growth factors switches on the phosphoinositide 3‐kinase (PI3K) signaling pathway, leading to the phosphorylation of phosphatidylinositol‐4,5,‐bisphosphate (PIP2) to phosphatidylinositol‐3,4,5,‐trisphosphate (PIP3) 27. Akt, another serine/threonine kinase, then binds to PIP3 and phosphorylates the upstream regulator of mTOR, tuberous sclerosis complex 2 (TSC2), which inactivates TSC and activates the mTORC1 complex, whose established substrates are S6K1 and 4EBP1, which control translation 27. Phosphatase and tensin homologue (PTEN), another upstream regulator of mTOR, dephosphorylates phosphoinositide substrates, thereby impairing Akt and mTOR activation 27, 28. Accordingly, compounds that specifically inhibit mTOR inhibit translation initiation, resulting in cell‐cycle arrest in the G1 phase of the cell cycle and potentially apoptosis 27, 29. Findings of aberrant mTOR signaling as an underlying factor in NET development, metastasis, and proliferation provide a rationale for use of mTOR inhibitors in the treatment of patients with NET.

### mTOR signaling and inhibition: clinical data

#### Everolimus as monotherapy

Efficacy of everolimus as monotherapy in patients with advanced pNET was evaluated in the randomized phase 3 RADIANT‐3 study that compared everolimus monotherapy with placebo 22. The primary end point was PFS, so crossover was allowed to address ethical and recruitment considerations. PFS per adjudicated central review was 11.4 months with everolimus versus 5.4 months with placebo, resulting in reduced risk (65%) for disease progression or death with everolimus treatment. Additionally, 64% of patients receiving everolimus achieved some degree of tumor shrinkage (including minor responses) versus 21% of patients receiving placebo. Notably, everolimus demonstrated a clinically and statistically significant prolongation of PFS regardless of previous chemotherapy 36. Based on results from RADIANT‐3, everolimus was FDA‐approved specifically for patients with progressive advanced pNET 18. OS data from RADIANT‐3 were recently reported. Improvement in median OS was shown with everolimus (median OS, 44.02 months; 95% confidence interval [CI], 35.6–51.8) versus placebo (median OS, 37.7 months; 95% CI, 29.1–45.8) (hazard ratio [HR], 0.94; 95% CI, 0.73–1.20; *P *=* *0.30; significance boundary, 0.0249) 37. However, the large number of patients in the placebo arm who crossed over to open‐label treatment might have influenced OS outcomes. Rank‐preserving structural failure time (RPSFT) analysis was performed to estimate the treatment effect corrected for crossover bias. RPSFT analysis showed a relative survival benefit of 3.27 (95% CI, 0.10–13.93) with survival rates of 82.6% versus 74.9% and 67.7% versus 55.6% at 12 and 24 months, respectively, for everolimus versus placebo 38.

Everolimus monotherapy in patients with other NET types is also being investigated in several ongoing trials. A phase 2 study in Europe, RAMSETE, investigated everolimus in patients with advanced (unresectable or metastatic), biopsy‐proven, nonfunctional NET with radiologic documentation of progressive disease (PD) with ≤3 prior systemic treatments. Of 73 patients enrolled, best response with monotherapy everolimus was stable disease (SD) in 55% 39. Median PFS was 185 days (95% CI, 158–255) 39. Moreover, disease stabilization by everolimus was demonstrated in all patient subgroups, including stratification by previous therapy 39 and primary tumor location 40.

A double‐blind, phase 3 study evaluated everolimus versus placebo (each plus best supportive care) in patients with advanced, nonfunctional gastrointestinal or lung NET with radiologically documented PD within 6 months and ≤1 prior systemic therapy (RADIANT‐4; NCT01524783). Several aspects of the RADIANT‐4 study design minimize the likelihood of confounding in the estimation of treatment effects, such as the non‐crossover design prior to the primary analysis, no use of concomitant SSAs, and prospective stratification of patients based on known prognostic factors (WHO performance status, tumor primary site, prior SSA exposure). The primary end point of this study was met, with a statistically significant and clinically meaningful 52% reduction in the relative risk of progression or death with everolimus versus placebo (HR 0.48 [95% CI, 0.35–0.67]; *P *<* *0.00001), and a median PFS of 11.0 and 3.9 months, respectively 41. This observed treatment benefit was maintained across all major subgroups, including lung and gastrointestinal NET (HR 0.50 [95% CI, 0.28–0.88] and HR 0.56 [95% CI, 0.37–0.84], respectively) 41. Based on this study, everolimus was approved by the FDA for the treatment of progressive, advanced, nonfunctional gastrointestinal and lung NET 18.

A phase 2 study is currently recruiting participants with pNET who were previously treated with surgery (R0 or R1) for liver metastases (NCT02031536). The primary objective of this study is to evaluate if the addition of adjuvant everolimus will result in an improvement in disease‐free survival, and the estimated primary completion date is October 2016.

#### Other agents targeting mTOR as monotherapy

In contrast to antitumor efficacy seen with everolimus, temsirolimus (Pfizer Inc), an intravenously administered mTOR inhibitor, did not demonstrate similar clinical benefit as a monotherapy among patients with advanced NET 42.

#### Combination therapy

A key rationale behind combining agents includes the potential for drugs to act through distinct mechanisms to overcome resistance, to act synergistically, and/or to improve efficacy. A potential might also exist to reduce dosages of individual agents, thereby improving tolerability. Safety profile is a critical factor with combination therapy. Agents with nonoverlapping toxicities are best suited for combination regimens because this allows the possibility of administering each agent at its maximum tolerated dose (MTD) 43. However, agents with broad target specificity, such as the TKI sunitinib, might be less suitable for combination therapy given the enhanced toxicity stemming from cumulative target and off‐target inhibition 43. For example, a high proportion of patients with renal cell carcinoma (RCC) and other solid tumors who were treated with sunitinib plus bevacizumab (Genentech, Inc., South San Francisco, CA) experienced grade 3/4 adverse events (AEs), including fatigue, hypertension, proteinuria, hand‐foot skin reaction, thrombocytopenia, and hemorrhage, and high rates of treatment discontinuation, dose reduction, or both, that were sufficiently high to offset clinical benefit 44.

Agents with more selective target specificities might therefore provide more flexibility for tailoring combination regimens to block specific molecular pathways relevant to individual patients based on the molecular profile of the tumor. In addition, toxicities might be more predictable or manageable because of limited off‐target effects 45. For example, in a phase 2 study in patients with advanced RCC, treatment with everolimus plus bevacizumab was associated with moderate rates of grade 3/4 AEs that were consistent with the known AE profiles associated with the individual agents 46.

Treatment with everolimus or temsirolimus in combination with other targeted agents has been investigated in patients with NET. The rationale is generally based on mechanism of action and supporting preclinical evidence. For example, the SSA octreotide improves NET‐related hormonal symptoms 47, 48, 49. Binding of octreotide to a somatostatin receptor (SSTR) inhibits hormones and other bioactive peptides and amines secreted by NET and has direct antiproliferative effects on tumor cells 50. SSTR_2_, the predominant SSTR subtype in NET, has a high binding affinity for octreotide 51. In both the RADIANT‐1 and RADIANT‐2 studies, it was hypothesized that combination therapy with everolimus plus octreotide LAR might enhance antitumor efficacy by simultaneously targeting upstream and downstream components of the mTOR pathway, because autocrine activation of the mTOR signaling pathway has been associated with NET cell proliferation 52 and mTOR inhibition has shown antiproliferative effects in NET 31, 48, 53. Clinical efficacy of everolimus plus octreotide LAR in patients with advanced NET was demonstrated in the phase 2 RADIANT‐1 trial 54. Promising results from RADIANT‐1 led to the subsequent phase 3 RADIANT‐2 study, in which efficacy of combined everolimus and an SSA was evaluated in 429 patients with low‐grade or intermediate‐grade NET. The addition of octreotide LAR to everolimus led to a trend toward improved PFS, with a 23% reduction in the estimated risk for progression (median PFS, 16.4 months vs. 11.3 months for placebo plus octreotide LAR; *P *=* *0.026) 48. Notably, this combination was associated with measurable indicators of efficacy, including reduction in tumor size, disease stabilization, and significant decreases in NET biomarker levels. However, the trial did not meet the prespecified threshold for statistical significance of the primary end point of PFS 48. Everolimus was generally well tolerated; frequently reported drug‐related AEs included stomatitis, rash, and diarrhea, consistent with phase 2 results 48.

A retrospective analysis based on the previous SSA exposure status of patients enrolled in the RADIANT‐2 trial revealed that the activity of combination therapy may depend on whether the patient's tumor is refractory to SSA 55. Patients who previously received SSA treatment had shorter median PFS than did SSA‐naïve patients (14.3 vs. 25.2 months, respectively; HR 1.44 [95% CI, 0.88–1.36]; *P *=* *0.140).

The combination of antiangiogenic agents such as the anti‐VEGF monoclonal antibody bevacizumab with everolimus might also be a feasible approach because the VEGF signaling pathway acts through the PI3K/mTOR pathway (Fig. 1), and the PI3K pathway is critical for endothelial cell activation and tumor angiogenesis 56. Thus, combining bevacizumab with mTOR inhibition might maximize inhibition of tumor angiogenesis. This combination allows the targeting of different components feeding into the PI3K/mTOR signaling pathway. A phase 2 study (NCT00607113) demonstrated antitumor activity of everolimus plus bevacizumab in patients with low‐grade or intermediate‐grade NET. The primary end point was functional biomarkers. Perfusion computed tomography was used as a functional biomarker of efficacy 57. Addition of everolimus to bevacizumab monotherapy resulted in greater decreases in tumor blood flow than bevacizumab alone (15%; *P *=* *0.02), and addition of bevacizumab to everolimus monotherapy led to a 21% decrease in tumor blood flow (*P *=* *0.01). At 6 months, median PFS was 14.4 months (95% CI, 12.7–16.1). OS rates at 12 and 24 months were 92% and 87%, respectively (median OS was not reached) 57.

In another randomized phase 2 trial, CALBG 80701 (NCT01229943) efficacy of everolimus plus bevacizumab was assessed versus everolimus alone in 150 patients with locally advanced or metastatic pNET; the primary end point was PFS 58. Median PFS was 16.7 months with combination treatment versus 14.0 months with monotherapy (HR, 0.80; 95% CI, 0.55–1.17; 116 PFS events; 1‐sided *P *=* *0.12). Median OS was 36.7 months with combination treatment versus 35.0 months with monotherapy (HR, 0.75; 95% CI, 0.42–1.33; 49 OS events; 1‐sided *P *=* *0.16). Treatment with everolimus plus bevacizumab was associated with a significantly higher response rate (31%) versus everolimus alone (12%; *P *=* *0.005). While this superior PFS is proof of concept for enhanced efficacy with combination therapy, it was associated with a higher rate of AEs. Temsirolimus in combination with bevacizumab has also been investigated for treatment of patients with well‐ or moderately differentiated pNET. Per preliminary results from a phase 2 study of 56 patients, the response rate was 41%. Median PFS and median OS were 13.2 months and 34.0 months, respectively 59.

Additional findings from recently presented data evaluating everolimus or temsirolimus plus various targeted agents are encouraging. In a phase 1 study in patients with advanced gastrointestinal or pNET, 200 mg twice daily of sorafenib (Bayer HealthCare Pharmaceuticals Inc., Whippany, NJ), the broadly targeted VEGFR‐TKI, plus 10 mg daily of everolimus was the MTD for this combination. Per independent review of best objective response, tumor shrinkage (including minor responses) was shown in 62% of patients 60. A phase 1 clinical trial evaluated temsirolimus plus vinorelbine (Pierre Fabre, Parsippany, NJ), a vinca alkaloid that interferes with microtubule assembly and disrupts cell division 61, 62. This trial enrolled 19 patients with advanced solid tumors, including one with pNET. The MTD with this combination was temsirolimus 25 mg (administered on days 1, 8, 15, and 22) and vinorelbine 20 mg/m^2^ (administered on days 1 and 15) in 4‐week cycles. Best response was partial response (PR) (*n* = 2) and SD (*n* = 8); median response duration was 3.2 months 62.

**Figure 2:**
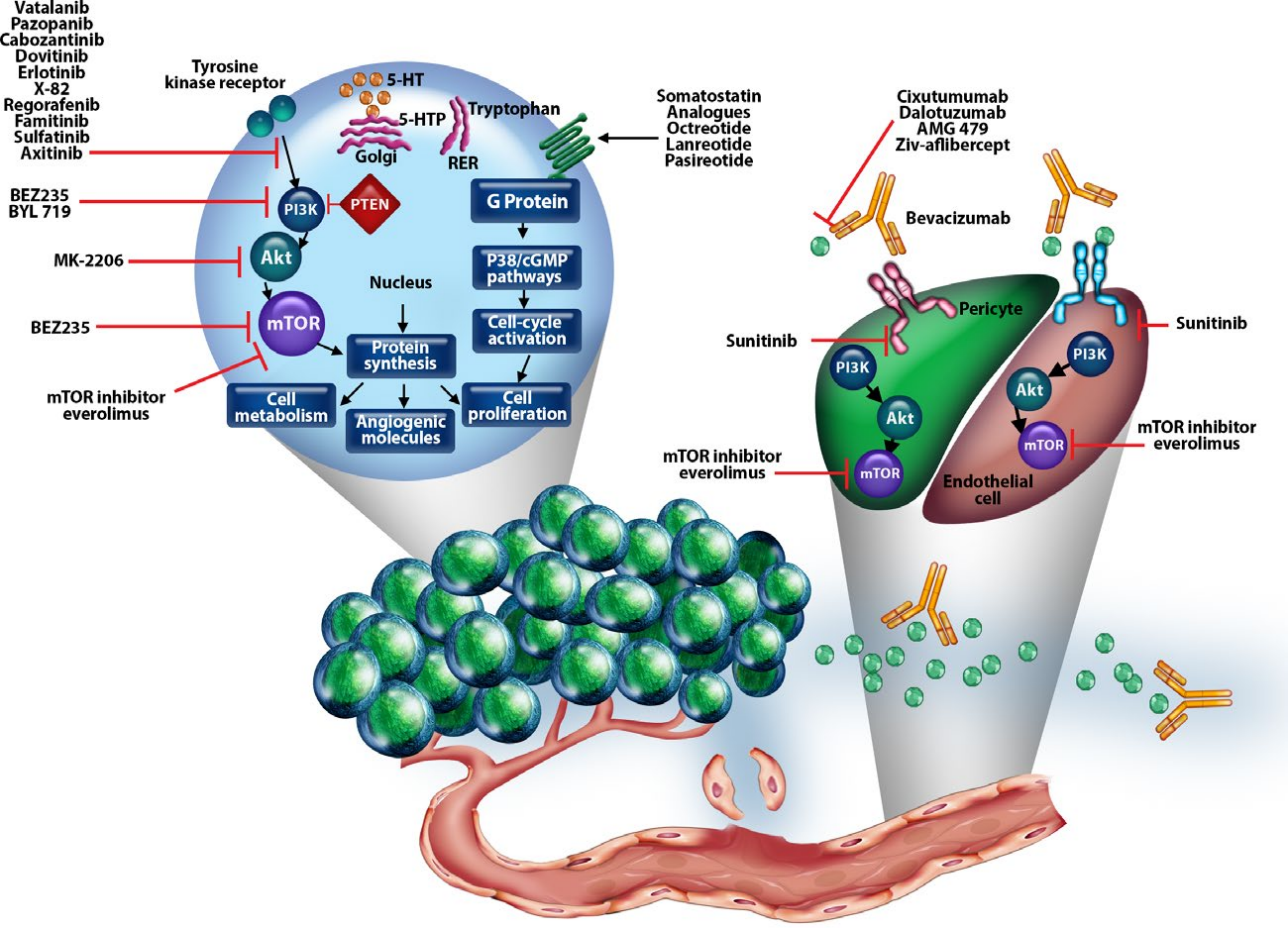
Targeted agents investigated in combination with mTOR inhibitors in patients with NET. Adapted from: Dong et al., New strategies for advanced neuroendocrine tumors in the era of targeted therapy 63, with permission from AACR.

A growing number of ongoing studies are evaluating other agents in combination with mTOR inhibitors in patients with NET (Table 1); some of these agents target components within the mTOR pathway or pathways that lead to mTOR activation (Fig. 2) 63. Because many act through complementary mechanism(s) of action, these agents provide a rationale for combination with blockade of the mTOR pathway 14, 64, 65, 66, 67, 68, 69, 70, 71, 72, 73, 74, 75, 76, 77. Among these is the SSA pasireotide long‐acting release (Novartis Pharmaceuticals Corporation), which has broader biologic activity than octreotide LAR or lanreotide Autogel, which preferentially bind to SSTR_2_ and SSTR_5_ and have moderate affinity for SSTR_3_ 50. Pasireotide targets SSTR_1,2,3_ and SSTR_5_ with high affinity 50, 67. Pasireotide long‐acting release has been demonstrated to have equivalent efficacy to high‐dose octreotide LAR for symptom control in patients with refractory carcinoid syndrome 78. Other combination therapy examples with mTOR inhibitors include erlotinib (Genentech, Inc.), an RTK targeting the EGFR 69 (NCT00843531); cixutumumab (Eli Lilly and Company, Indianapolis, IN), a fully humanized anti‐IGF 1 receptor (IGF‐1R) monoclonal antibody 73 (NCT01204476); and vatalanib (Cayman Chemical, Ann Arbor, MI), which inhibits VEGFR_1‐3_ 70 (NCT00655655). In addition to targeted agents, the alkylating agent temozolomide has demonstrated preliminary activity in combination with everolimus and acceptable tolerability in patients with pNET 79. In a phase 1/2 study of 43 patients with low‐ or intermediate‐grade pNET receiving everolimus plus temozolomide, this combination showed acceptable safety; the most commonly occurring grade 3 or 4 AEs were anticipated hematologic events. Results were considered promising—of 40 patients evaluable for response, 16 had PR (40%), 21 had SD (53%), and 3 had PD (7%) 79 (NCT00576680). A phase 2 study in patients with advanced grade 3 gastroenteropancreatic NET (NCT02248012) evaluating this combination is currently in progress.

**Table 1:** Select ongoing studies evaluating combination therapies of mTOR inhibitors and other agents

Agent(s) in combination with mTOR inhibitors	Study	Type of NET	Mechanism of action of non‐everolimus component
Everolimus + pasireotide long‐acting release	Open‐label, phase 1 COOPERATE‐1 study; *N* = 36; NCT01263353; completed	Advanced pulmonary or gastrointestinal NET	Pasireotide: SSA Mimics natural somatostatin 67; inhibits GH, cortisol, IGF‐1, and other hormones secreted in carcinoid tumors 67; controls symptoms such as diarrhea and flushing 68
	Open‐label, phase 1 extension of COOPERATE‐1 study; *N* = 17; NCT01590199; ongoing, not recruiting	Advanced pulmonary or gastrointestinal NET	
	Open‐label, phase 1 trial; *N* = 32; NCT00804336; ongoing, not recruiting	Advanced NET	
	Open‐label, phase 2 COOPERATE‐2 study; *N* = 160; NCT01374451; completed	Advanced pNET	
	Randomized, 3‐arm, phase 2 LUNA study; *N* = 124; NCT01563354; ongoing, not recruiting	Advanced pulmonary or thymus NET	
Everolimus + erlotinib	Open‐label, phase 2 trial; *N* = 17; NCT00843531; ongoing, not recruiting	Well‐ to moderately differentiated NET	Erlotinib: EGFR inhibitor 69
Everolimus + temozolomide	Open‐label, phase 2 study; *N* = 40; NCT02248012; recruiting	Advanced, grade 3 gastroenteropancreatic NET	Temozolomide: Alkylating agent 66; delivers a methyl group to guanine bases of DNA 66
Everolimus + vatalanib	Phase 1 study; *N* = 96; NCT00655655; ongoing, not recruiting	Advanced solid tumors, including NET	Vatalanib: Inhibits VEGFR1‐3, PDGFR‐*β*, c‐KIT, and c‐FMS 70
Everolimus + X‐82	Open‐label, phase 1/2 study; *N* = 71; NCT01784861; recruiting	Advanced solid tumors (phase 1), unresectable or metastatic, well‐ or moderately differentiated pNET (phase 2)	X‐82: Dual VEGFR/PDGFR inhibitor 74
Everolimus + BYL719	Open‐label, phase 1b study; *N* = 166; NCT02077933; recruiting	Advanced solid tumors, with dose expansion in pNET	BYL719: Selective PI3K*α* inhibitor 75
Everolimus followed or preceded by streptozocin + fluorouracil	Randomized, open‐label phase 3 study; *N* = 180; NCT02246127; recruiting	Advanced, well‐differentiated pNET	Streptozocin: DNA alkylating agent 64Fluorouracil: Antimetabolite 76
Everolimus + SNX 5422	Open‐label, phase 1 study; *N* = 15; NCT02063958; recruiting	Advanced NET of gastroenteropancreatic or pulmonary origin	Inhibitor of heat shock protein 90 77
Temsirolimus + bevacizumab	Open‐label phase 2 study; *N* = 299; NCT01010126; Ongoing, not recruiting	Locally advanced, recurrent, metastatic, or progressive pNET or carcinoid tumor	Bevacizumab: Anti‐VEGF monoclonal antibody 72; inhibits angiogenesis 72

c‐KIT, a receptor tyrosine kinase (type of tumor marker and stem cell factor receptor, also known as CD117); EGFR, epidermal growth factor receptor; GH, growth hormone; IGF‐1, insulin‐like growth factor 1; IGF‐1R, insulin‐like growth factor 1 receptor; mTOR, mammalian target of rapamycin; NET, neuroendocrine tumors; PDGFR, platelet‐derived growth factor receptor; pNET, pancreatic neuroendocrine tumors; SSA, somatostatin analog; VEGFR, vascular endothelial growth factor receptor.

### Novel agents along the mTOR pathway

Various novel agents are being evaluated for the treatment of patients with NET, including second generation mTOR inhibitors that use a multitargeted inhibition approach with the potential to overcome tumor escape mechanisms. For example, the dual PI3K/mTOR (mTORC1 and mTORC2) inhibitor BEZ235 (Selleck Chemicals, Houston, TX) has been found to prevent feedback activation of Akt in NET cell lines, a well‐known AE of single mTOR inhibition that has been suggested to attenuate the antitumor efficacy of mTOR inhibition 80, 81. A phase 2 study evaluated BEZ235 in 31 patients with advanced pNET who progressed on treatment with everolimus. SD was achieved by 51.6% of patients after 16 weeks of treatment. However, many patients discontinued treatment because of AEs 82. The benefits versus safety risks associated with such combination treatment warrant further study, as multitargeted inhibition appears to be a promising approach in NET treatment.

Studies evaluating monoclonal antibodies targeting IGF‐1R (including dalotuzumab [MK‐0646; Merck & Co., Inc., Kenilworth, NJ] 83, 84, and AMG‐479 [Amgen Inc, Thousand Oaks, CA] 85) in patients with NET have shown limited promise for those agents 83, 85, 86. A phase 1, single‐institution study evaluated the recommended phase 2 dose (RP2D) for the combination of cixutumumab, everolimus, and octreotide LAR in patients with WD‐NET. The RP2D of this combination was found to be cixutumumab 10 mg/m^2^, octreotide LAR 20 mg IM q 21 days, and everolimus 10 mg daily 87.

Moving beyond the combination of mTOR and either SSAs or VEGF pathway inhibitors is the novel concept of overcoming drug resistance. All drug therapies have resistance mechanisms. For example, a potential escape mechanism for everolimus might involve upregulation of PI3K and other pro‐survival pathways 81, 88, 89, 90. Interestingly, previous reports have shown that autophagy is upregulated on mTOR inhibition in various cancers. Therapy used for pancreatic cancer xenografts and mouse models with autophagy inhibitors has been demonstrated to cause tumor regression and extend survival 91. The most commonly used drug to inhibit autophagy has been chloroquine and its active derivative hydroxychloroquine, which affect late‐stage autophagy 92, 93, 94. Novel autophagy inhibitors such as *N*‐acetyl cysteine and 3‐methyladenine, which have been approved by the FDA for other diseases, might affect autophagy at an earlier stage and be more effective therapeutically 92.

## Conclusions

mTOR and the many proteins involved in this signaling pathway play a central role in the life cycle of neuroendocrine carcinomas. Agents that target multiple components of this pathway are potentially valuable in improving treatment outcomes in patients with NET. Better understanding of the molecular mechanisms of resistance to inhibitors of mTOR will further assist with the development of future preclinical and clinical studies. Ongoing investigations of novel combination regimens using both approved and investigational agents will reveal which of these treatment options will provide greater benefit in patients with NET. The mTOR inhibitor everolimus is currently approved for lung and gastrointestinal NET and pNET, but shows promise for use in the adjuvant setting and as combination therapy. The identification of biomarkers predictive of mTOR inhibitor response is a future challenge for the treatment of patients with NET.

## Conflict of Interest

None declared.
